# The role of CEA, CYFRA21-1 and NSE in monitoring tumor response to Nivolumab in advanced non-small cell lung cancer (NSCLC) patients

**DOI:** 10.1186/s12967-019-1828-0

**Published:** 2019-03-08

**Authors:** M. G. Dal Bello, R. A. Filiberti, A. Alama, A. M. Orengo, M. Mussap, S. Coco, I. Vanni, S. Boccardo, E. Rijavec, C. Genova, F. Biello, G. Barletta, G. Rossi, M. Tagliamento, C. Maggioni, F. Grossi

**Affiliations:** 1 Lung Cancer Unit, IRCCS-Ospedale Policlinico San Martino, Genova, Italy; 2 Clinical Epidemiology Unit, IRCCS-Ospedale Policlinico San Martino, Genova, Italy; 3 Nuclear Medicine Unit, IRCCS-Ospedale Policlinico San Martino, Genova, Italy; 4 Laboratory Medicine Unit, IRCCS-Ospedale Policlinico San Martino, Genova, Italy; 5 Department of Internal Medicine and Medical Specialties (DIMI), University of Genova, Genova, Italy; 6 Division of Medical Oncology, Fondazione IRCCS Ca’ Granda Ospedale Maggiore Policlinico, Milano, Italy

**Keywords:** NSCLC, CYFRA21-1, CEA, Immunotherapy, Tumor response, Survival

## Abstract

**Background:**

CEA, CYFRA21-1 and NSE are tumor markers used for monitoring the response to chemotherapy in advanced adenocarcinoma, squamous cell carcinoma and small-cell lung cancer, respectively. Their role in cancer immunotherapy needs to be elucidated.

**Methods:**

Patients with advanced non-small cell lung cancer (NSCLC) were treated with nivolumab 3 mg/kg every 2 weeks within the Italian Nivolumab Expanded Access Program. Blood samples were collected at baseline, at each cycle up to cycle 5 and then every two cycles until patient’s withdrawn from the study. All patients underwent a CT-scan after every 4 cycles of treatment and responses were classified according to RECIST 1.1. The biomarkers serum levels were measured with a chemiluminescent microparticle immunoassay for CEA and with an immuno radiometric assay for CYFRA21-1 and NSE. The markers values at baseline and after 4 cycles were used to analyze the relationship between their variation over baseline and the tumor response, evaluated as disease control rate (DCR: CR + PR + SD), and survival (PFS and OS).

**Results:**

A total of 70 patients were evaluable for the analysis. Overall, a disease control was obtained in 24 patients (35.8%, 4 PR + 20 SD). After 4 cycles of nivolumab a CEA or CYFRA21-1 reduction ≥ 20% over the baseline was significantly associated with DCR (CEA, p = 0.021; CYFRA21-1, p < 0.001), PFS (CEA, p = 0.028; CYFRA21-1, p < 0.001) and OS (CEA, p = 0.026; CYFRA21-1, p = 0.019). Multivariate analysis confirmed the ability of CYFRA21-1 reduction ≥ 20% to predict DCR (p = 0.002) and PFS (p < 0.001).

**Conclusion:**

The reduction in serum level of CYFRA21-1 or CEA might be a reliable biomarker to predict immunotherapy efficacy in NSCLC patients. NSE was not significant for monitoring the efficacy of nivolumab.

## Background

Advanced lung cancer remains the leading cause of cancer related deaths worldwide being the treatment of disease still challenging [1]. Immunotherapy is a standard of treatment in advanced non-small cell lung cancer (NSCLC) patients progressing after a first-line chemotherapy or as first-line treatment in combination with chemotherapy or as single agent in patients with high expression of PD-L1. Several agents targeting immune checkpoints have been tested with remarkable results on survival and manageable toxicity [2]. Nivolumab (BMS-936558) is a fully human IgG4 programmed cell death 1 (PD-1) immune checkpoint inhibitor that enhances the immune T cell response by blocking the interaction between the PD-1, an inhibitory receptor on activated T lymphocytes, and the programmed cell death ligand 1 (PD-L1) expressed on cancer cells. Two randomized Phase III studies have been reported on squamous (CheckMate 017) and non-squamous (CheckMate 057) NSCLC [3, 4] leading to drug approval by the Food and Drug Administration (FDA) and the European Medicines Agency (EMA) for advanced or metastatic NSCLC after prior chemotherapy. This improvement in the management of advanced NSCLC has required the identification of prognostic and/or predictive biomarkers to select the best candidates to immunotherapy and to monitor the tumor response [5]. PD-L1 expression has been widely explored as a potential marker but its role in the clinical setting is still controversial [6]. Serological biomarkers such as carcinoembryonic antigen (CEA), cytokeratin fragment 19 (CYFRA21-1) and neuron-specific enolase (NSE), have been mainly investigated as prognostic or predictive markers in NSCLC patients treated with chemotherapy [7, 8]. CEA is a serum glycoprotein and currently is the most widely used marker for colorectal, breast and lung cancer. Increased levels of CEA are observed in smokers and in presence of non-neoplastic disease [9, 10]. CYFRA21-1 is a fragment of cytokeratin 19 that is abundant in the pulmonary tissue. Serum concentrations are particularly elevated in the carcinoid tumors and in squamous cell carcinoma of the lung where it correlates with the tumor size, lymph node status and the stage of disease [11, 12]. As a result, CEA and CYFRA21-1 have been identified as useful prognostic factors [7–13], as predictors of efficacy for targeted therapy [14, 15] or chemotherapy [8] and as markers of postoperative recurrence and metastasis [16–18]. NSE is a cytosolic enzyme expressed at high levels in the brain and preferentially in neurons and neuroendocrine cells [19]. As a specific serum marker of neuronal injury, elevated levels of NSE have been found in cancers of neuroendocrine cellular origin, including small-cell lung cancer (SCLC) where it correlates with the extent of disease [20, 21]. For SCLC the NSE has a specificity around 85% and is useful for prognosis of survival, monitoring of treatment and prediction of relapse [16, 21, 22]. Increased levels of NSE have also been reported in NSCLC where its role as predictive and prognostic marker is still under debate. Tiseo et al. reported a significant correlation between higher baseline serum NSE levels and response to standard first-line chemotherapy in advanced NSCLC whereas did not find a prognostic role [23]. A recent meta-analysis including 2389 NSCLC patient has confirmed the lack of prognostic significance for NSE [24]. In addition, in a recent study Fiala et al. have showed a negative predictive role of high baseline NSE levels in NSCLC patients treated with epidermal growth factor tyrosine kinase inhibitors (EGFR-TKIs) [25]. The role of CEA, CYFRA21-1 and NSE in monitoring the response to immunotherapy in NSCLC patients needs to be elucidated. In the present study we tested the hypothesis that their variation compared to the baseline may act as indicators of treatment efficacy and survival in advanced NSCLC patients treated with nivolumab.

## Methods

### Patient’s enrollment

Between May 2015 and May 2016, 74 consecutive patients with advanced NSCLC previously treated with at least one line of chemotherapy were prospectively enrolled in a single-institutional translational research study at the Ospedale Policlinico San Martino in Genova, Italy, within the Italian Nivolumab Expanded Access Program. This study was approved by the Ethics Committee of Liguria Region (Italy) (P.R.191REG2015) and conducted in compliance with the principle of the Declaration of Helsinky; a written informed consent was acquired from all patients. All the patients were treated with nivolumab at the dose of 3 mg/kg every 2 weeks until disease progression, unacceptable toxicity, patient refusal, or death. Baseline assessments were done with a computed tomography scan (CT scan) of the chest and abdomen within 2 weeks before treatment and then after 4 cycles of treatment. The tumor response was assessed according to Response Evaluation Criteria in Solid Tumors (RECIST ver 1.1) [26]. Each patient’s response was classified into one of the following categories: responders, including case of complete response (CR), partial response (PR) and stable disease (SD), and non-responders including cases of disease progression (PD). Disease control rate (DCR) was defined as those patients who had obtained a CR, a PR or a SD. For patients who achieved a PD, an additional assessment was performed after 2 further cycles to confirm PD; if PD was confirmed, treatment was discontinued.

### Specimen collection and tumor marker assays

The tumor markers were determined collecting a blood sample before treatment initiation (baseline visit), at each cycle up to cycle 5 and then every two cycles until patient’s withdrawn from the study. Serum levels of CEA were detected using a commercially available chemiluminescent microparticle immunoassay (Architect CEA Reagent kit, Abbott Diagnostics Division) whereas CYFRA21-1 and NSE were detected using a commercially available immuno radiometric assay (Cytokeratin 19 Fragment IRMA Kit and NSE IRMA Kit, Beckman Coulter Inc.) according to the manufacturer’s instructions. The reference range was 0 to 5 ng/ml for CEA, 0 to 3.3 ng/ml for CYFRA 21-1, 0 to 13.4 ng/ml for NSE. Hemolyzed samples were excluded from the analysis. The markers levels at baseline and after 4 cycles of nivolumab were used to analyze the relationship between their variation over the baseline and the tumor response, considered as disease control rate (DCR), progression free survival (PFS) and overall survival (OS). On the basis of the results from an our previous study in advanced NSCLC patients treated with standard first-line chemotherapy [8], a post-treatment drop in serum concentration ≥ 20% over baseline was used as cut-off level for defining a marker response. In addition, a sub-analysis of the three markers in the different histological types was further investigated.

### Statistical analysis

Variables were summarized as median (range) for continuous variables and number (%) for categorical variables. Relationships between categorical variables were examined by means of the Chi square test. Patients were categorized according to median age (≤ 70 and > 70 years) and histology (adenocarcinoma vs squamous cell carcinoma). Non-parametric tests were used to check differences between the two groups and to compare the markers values at baseline and after 4 cycles of treatment. Odds Ratios (OR) and the corresponding 95% confidence intervals (95% CI) for a set of individual and clinical variables were computed to predict therapy response in a multiple logistic analysis. Univariate and multivariate analyses were performed to evaluate the prognostic impact on PFS and on OS; PFS was calculated from the start of nivolumab to the date of PD or death or last follow-up; OS was calculated from the start of nivolumab to the date of death or last follow-up. The Kaplan–Meier method was applied to estimate survival probabilities and the log-rank test was carried out to assess heterogeneity within each prognostic factor. Cox’s proportional hazards regression model was carried out as multivariate analysis to assess the prognostic role of the markers adjusted for the possible confounding effect of all other factors included in the same model. All statistical test were two-sided, and variables that had *p*-values of less than 0.05 were considered significant.

## Results

### Patients and tumor characteristics

Seventy out of 74 patients were evaluable for serum markers and response assessment after 4 cycles of nivolumab (4 patients were excluded from the analysis for hemolyzed baseline samples). Three patients stopped nivolumab for toxicity before the first CT scan evaluation. The clinicopathological characteristics are summarized in Table 1. The median age was 70 years (range 44–85) and 69% of patients were male. NSCLC included 54 adenocarcinomas (77%), 15 squamous cell carcinomas (22%) and one case of not otherwise specified (NOS) type (1%). The majority of the patients were smokers (87%), had metastatic disease (96%) and ECOG PS 0–1 (92%). The median number of prior lines of treatment was 2 (range 1–6). The median value of the serum levels of the three markers at baseline (pre-treatment) was 6.6 ng/ml for CEA (range 0.8–2615), 5 ng/ml for CYFRA21-1 (range 0.2–126.4) and 7.5 ng/ml (range 3.1–46.8) for NSE. Pre-treatment values over the upper normal limit of CEA, CYFRA 21-1 and NSE were detected in 40 (57%), 45 (64%), and 14 (20%) patients, respectively. At cycle 2 and cycle 3 data on CEA were available for 59 and 54 patients, respectively, while data on CYFRA 21-1 were available for 57 and 54 patients, respectively. At the same time points data on NSE were available for 58 and 50 patients, respectively.

**Table 1:** Clinicopathological characteristics

	No. of patients (70)	%
Age, median (range, year)	70 (44–85)	
*Gender*		
Male	48	69
Female	22	31
*Histology*		
Adenocarcinoma	54	77
Squamous	15	22
NOS	1	1
*Stage*		
IIIB	3	4
IV	67	96
*ECOG PS*		
0	25	36
1	39	56
2	6	8
*Smoking habits*		
Never smoker	9	13
Former smoker	35	50
Smoker	26	37
*Prior lines of therapy*	Median 2 (range 1–6)	
1	28	40
2	20	29
3	13	19
≥ 4	9	12
*CEA (ng/ml) baseline*		
Median (range)	6.6 (0.80–2615)	
Normal (< 5)	30	43
Elevated (≥ 5)	40	57
*CYFRA 21*-*1 (ng/ml) baseline*		
Median (range)	5.0 (0.2–126.4)	
Normal (< 3.3)	25	36
Elevated (≥ 3.3)	45	64
*NSE (ng/ml) baseline*		
Median (range)	7.5 (3.1–46.8)	
Normal (< 3.3)	56	80
Elevated (≥ 3.3)	14	20

NOS, not otherwise specified; ECOG, Eastern cooperative oncology group; PS, performance status; CEA, carcinoembryonic antigen; CYFRA21-1, cytokeratin fragment 19; NSE, neuron-specific enolase

### Correlation between serum markers levels, clinic-pathologic features and tumor response

No significant correlation was found between baseline markers serum levels and age or gender. Abnormal baseline CEA levels were found in current smokers (p = 0.048) and in adenocarcinomas (p < 0.001). Abnormal, but not significant, baseline CYFRA21-1 levels were found in squamous tumors. No association was found between baseline NSE levels and patient and cancer characteristics (data not shown). On average, patients received 6 cycles of nivolumab (range 1–36) and a first CT scan evaluation was performed after a median time of 6.9 weeks, corresponding to 4 cycles of nivolumab,. Overall, a disease control was obtained in 24/67 patients (35.8%, 4 PR and 20 SD). Age, gender, histology, stage, ECOG PS, smoking habit and baseline serum levels did not correlate with response to nivolumab (data not shown). After 4 cycles of nivolumab the median CEA and NSE levels remained rather stable compared to baseline (5.1 ng/ml and 7.4 ng/ml, respectively) while the median CYFRA 21-1 levels dropped to 2.7 ng/ml. Interestingly, in those patients who obtained a DCR we observed a decline of all three serum markers with a significant difference between responders and no-responders (Table 2). Overall, CEA, CYFRA 21-1 and NSE reduction ≥ 20% occurred in 13/49 (26%), 17/50 (34%) and 16/44 (36%) patients, respectively, and a CEA and CYFRA 21-1 reduction were associated with favorable DCR (Table 3). With RECIST, a decrease ≥ 20% of CEA was achieved in 43.5% of responders and in 11.5% of no-responders (p = 0.021), while a decrease ≥ 20% of CYFRA21-1 occurred in 62.5% of responders and in 7.7% of no-responders (p < 0.001). Interestingly, we observed that a tumor response occurred in 87.5% of patients with a CYFRA21-1 reduction ≥ 20% already presents after the 1st cycle (p = 0.008) and in 80% of patients with a CEA reduction ≥ 20% already presents after the 2nd cycle (p = 0.033) (data not shown).

**Table 2:** CEA, CYFRA 21-1 and NSE variation according to response to nivolumab

	Median (%)	Range (%)	p-value
*CEA*			
No responder	+ 31	− 79; + 498	0.005
Responder	− 9	− 92: + 88	
*CYFRA21-1*			
No responder	+ 72	− 62; + 508	< 0.001
Responder	− 37	− 98; + 2220	
*NSE*			
No responder	+ 20	− 64; + 182	0.012
Responder	− 14	− 79; + 71

**Table 3:** Markers reduction ≥ 20% over baseline and tumor response (R)

	No-R n (%)	R n (%)	p-value
*CEA reduction ≥ 20%*			
No	23 (88.5)	13 (56.5)	0.021
Yes	3 (11.5)	10 (43.5)	
*CYFRA21-1 reduction ≥ 20%*			
No	24 (92.3)	9 (37.5)	< 0.001
Yes	2 (7.7)	15 (62.5)	
*NSE reduction ≥ 20%*			
No	17 (73.9)	11 (52.4)	0.21
Yes	6 (26.1)	10 (47.6)	

Multivariate analysis, including variables for age, gender, CEA and CYFRA reduction ≥ 20%, revealed that CYFRA21-1 reduction ≥ 20% was an independent positive predictor factor for DCR (HR 4.36, 95% CI 1.7 to 11.3, p = 0.002) (Table 4). Interestingly, we observed that the reduction ≥ 20% of the tumor markers was already evident at the beginning of the therapy. In particular, the decrease of at least 20% had already evident after the 1th, 2nd and 3rd cycle in 5%, 20%, and 28% of patients for CEA, in 15%, 37% and 39% of patients for CYFRA21-1 and in 26%, 22%, and 34% of patients for NSE, respectively (data not shown). Finally, analyzing the tumor markers on the basis of histotype we observed that patients with adenocarcinoma reached a DCR when CEA and CYFRA21-1 reduction was ≥ 20%, with a significant difference in response compared to the patients with marker reduction < 20% (CEA, 77% vs 40%, p = 0.043; CYFRA21-1, 92% vs 35%, p = 0.001). Among the patients with squamous cell carcinoma, we observed a reduction ≥ 20% for CYFRA21-1 and NSE but only a CYFRA21-1 reduction resulted in DCR (p = 0.033). In both histological types NSE reduction ≥ 20% did not show to be significantly associated with DCR (data not shown).

**Table 4:** Ability of CEA and CYFRA 21-1 to predict DCR (CR + PR + SD) in a multivariate analysis

	Odds ratio	95% CI	p-value
*Gender*			
Male	1.0		0.13
Female	1.85	(0.8–4.1)	
*Age*			
≤ 70	1.0		0.48
> 70	1.31	(0.6–2.8)	
*CEA reduction ≥ 20%*			
No	1.0		0.32
Yes	1.58	(0.6–3.9)	
*CYFRA 21-1 reduction ≥ 20%*			
No	1.0		0.002
Yes	4.36	(1.7–11.3)	

### Association between CEA, CYFRA 21-1, NSE and PFS

**Fig. 1:**
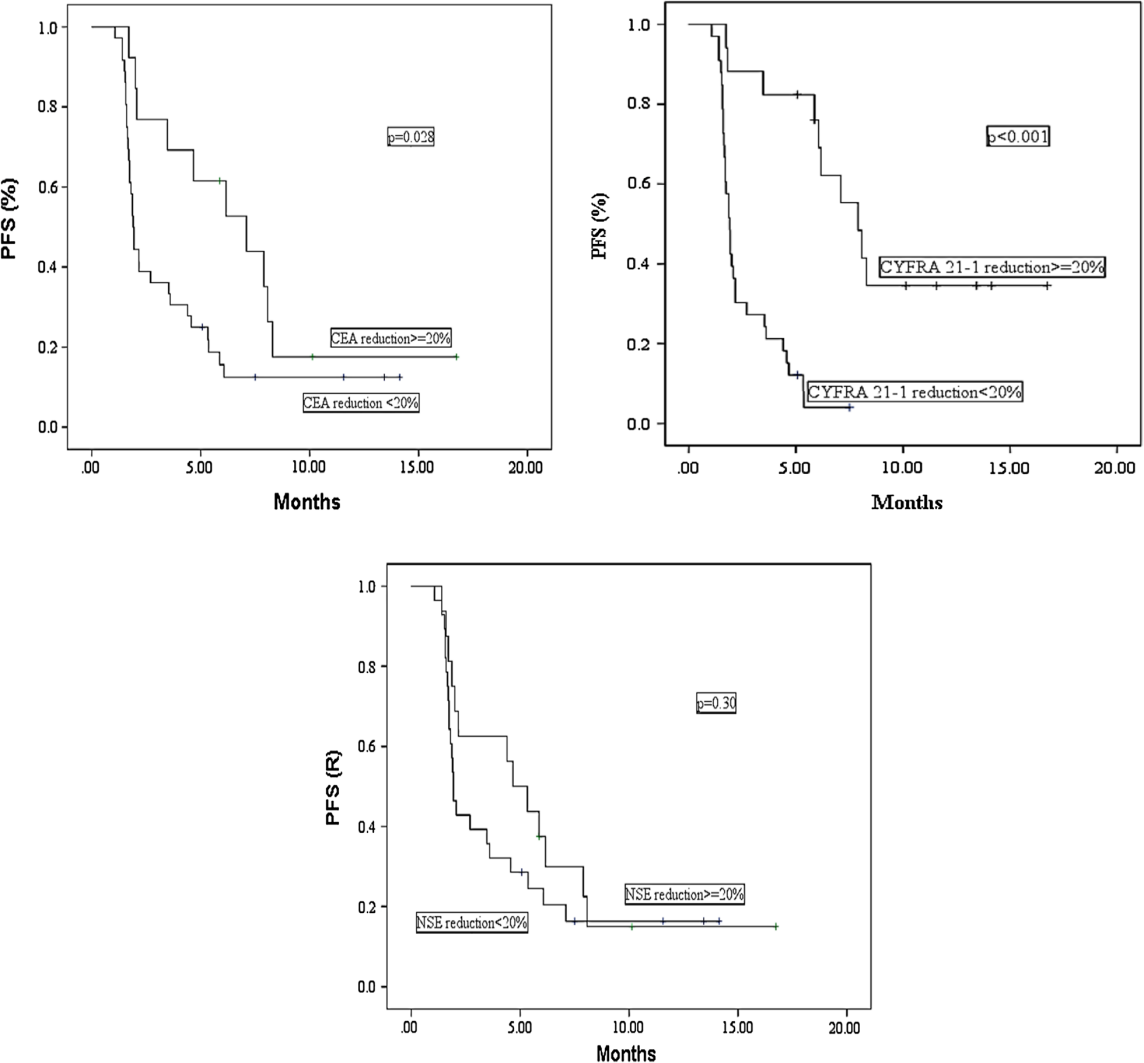
Progression-free survival according to CEA, CYFRA21-1 and NSE reduction ≥ 20%

Overall, median PFS on 67 patients was 1.9 months (95% CI 1.7–2.2 months). Age, sex, histology, PS, smoking, prior treatment lines and baseline serum marker levels were not associated with PFS. In contrast, a longer PFS was observed in patients with normal baseline CEA values (2.7 months vs 1.7 months, p = 0.026) and with a CEA and CYFRA21-1 reduction ≥ 20% after 4 cycles of nivolumab (CEA: 7.1 vs 1.9 months, p = 0.028; CYFRA21-1: 7.9 vs 1.9 months, p < 0.001). No significant association was found between NSE reduction ≥ 20% and PFS (4.7 vs 1.9 months, p = 0.300) (Fig. 1). Multivariate analysis including terms for gender, age, CEA, CYFRA21-1 and NSE reduction ≥ 20% confirmed the positive prognostic role only for CYFRA21-1 reduction ≥ 20% (HR = 0.35, 95% CI 0.20–0.60, p < 0.001) (data not shown). Considering histology, a marker’s reduction improved PFS in adenocarcinoma patients (CEA,7.1 vs 1.9 months, p = 0.013; CYFRA21-1, 7.9 vs 1.9 months p < 0.001; NSE 5.9 vs 1.9 months, p = 0.067), while in patients with squamous carcinoma PFS was improved only in patients with CYFRA21-1 reduction ≥ 20% (6.1 vs 1.7 months, p = 0.032).

### Association between CEA, CYFRA 21-1, NSE and OS

For the whole study population, median follow up was 10.7 months (range 5.0–16.8) for censored patients and 3.1 months (range 0.1–13.2) for deceased patients. The association between clinicopathological characteristics and serum markers with OS is shown in Table 5. Median survival time was 9.2 months (95% CI 5.3–13.2). During the study period, 40 patients (57.1%) died. In the univariate analysis, a statistically significant prognostic effect was found for number of prior lines of treatment (n = 1, 6.1 months, 95% CI = 3.6–8.5; n ≥ 2, 12.2 months, 95% CI = 8.2–13.3, p = 0.036) and for response to therapy (13.5 months for responders vs 6.4 months for no-responders, p < 0.001). At baseline, normal markers levels were significantly associated with better OS: 12.1 months for CEA < 5 ng/ml vs 5.6 months for CEA ≥ 5 ng/ml, p = 0.035; 13.2 months for CYFRA21-1 < 3.3 ng/ml vs 5.6 months for CYFRA21-1 ≥ 3.3 ng/ml, p = 0.005 and 10.0 months for NSE < 13.4 ng/ml vs 2.2 months for NSE ≥ 13.4 ng/ml, p = 0.028.

**Table 5:** OS according to clinicophathological characteristics

	Mean OS (95% CI)^a^ (months)	p-value
Overall	9.2 (5.3–13.2)	
*Age (years)*		
≤ 70	6.1 (0.3–11.8)	0.27
> 70	10.0 (7.2–12.8)	
*Gender*		
Male	8.9 (5.1–12.8)	0.76
Female	9.2 (2.3–16.1)	
*Histology*		
Adenocarcinoma	9.2 (4.6–13.9)	0.56
Squamous	9.8 (2.5–17.2)	
*PS ECOG*		
0	9.2 (5.6–12.8)	0.65
> 1	2.0 (0.1–5.4)	
*Smoke*		
Never smoker	9.9 (0.1–20.5)	0.80
Smoker	8.9 (4.7–13.2)	
*Prior treatment lines, n*		
1	6.1 (3.6–8.5)	0.036
≥ 2	12.2 (8.2–13.3)	
*RECIST response*		
No response	6.4 (4.8–8.0)	< 0.001
Response	13.5 (11.2–15.7)	

^a^Median survival not reached

**Fig. 2:**
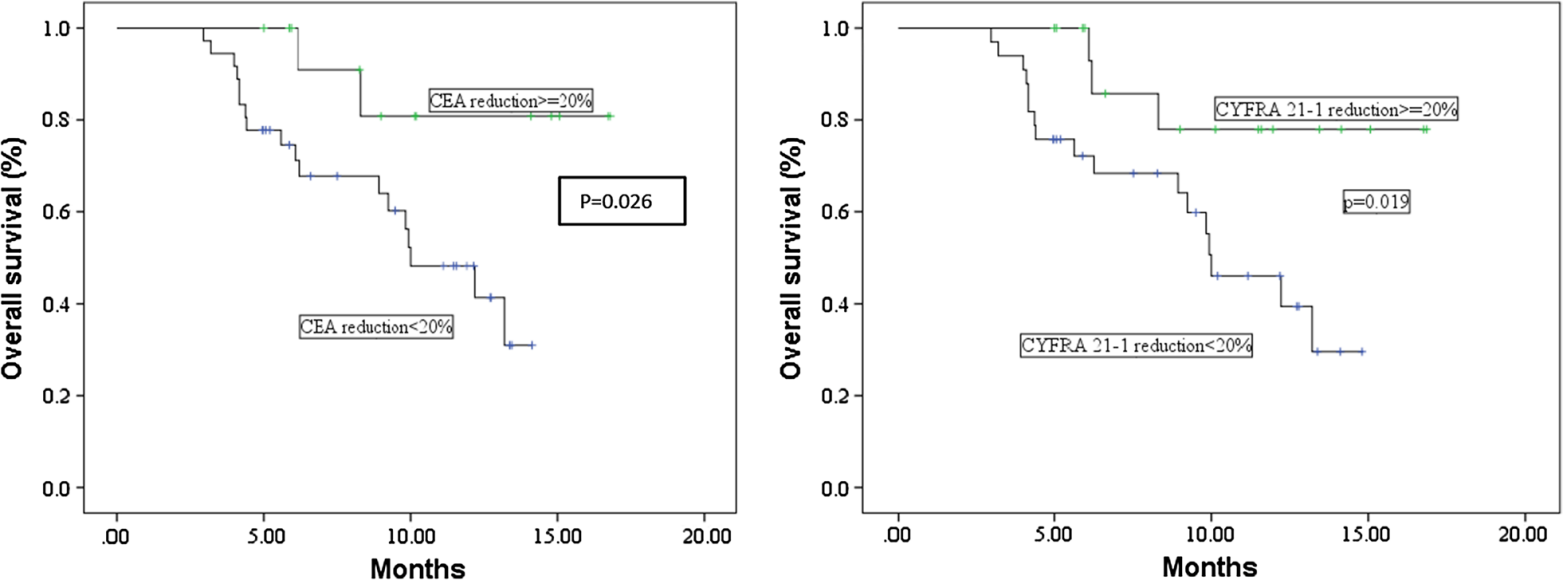
Overall survival according to CEA and CYFRA21-1 reduction ≥ 20%

In addition, also a reduction ≥ 20% of CEA or CYFRA21-1 after 4 cycles of nivolumab represented a positive prognostic factor (Table 6). In particular, Kaplan–Meier survival curves showed that patients with CEA or CYFRA21-1 reduction ≥ 20% survived longer than patients with no marker reduction (15 months vs 9.9 months, p = 0.026 and 14.6 months vs 10 months, p = 0.019, respectively) (Fig. 2). Multivariate analysis taking into account gender, age, prior lines of therapy and baseline CEA, CYFRA21-1 and NSE levels showed a better prognosis for patients with a higher number of therapies (≥ 2 lines: HR = 0.67, 95% CI 0.48–0.94, p = 0.022) and with normal baseline CEA or CYFRA21-1 levels (CEA ≤ 5 ng/ml: HR = 0.70, 95% CI 0.49–1.01, p = 0.057; CYFRA21-1 ≤ 3.3 ng/ml: HR = 0.68, 95% CI 0.46–1.01, p = 0.055). Multivariate analysis taking into account CEA and CYFRA21-1 reduction ≥ 20% did not show statistically significant results but a tendency towards a better prognosis for patients with a CYFRA21-1 reduction ≥ 20% (HR = 0.55, 95% CI 0.28–1.07, p = 0.079) (data not shown). Finally, with regard to histologic subtypes, no significant difference in OS was observed between patients with adenocarcinoma compared to squamous carcinoma (median OS, 9.2 vs 9.8 months). Of note, OS was significantly increased only among adenocarcinoma patients with CEA reduction ≥ 20% (median OS, 14.8 vs 9.9 months, p = 0.054) (data not shown).

**Table 6:** OS according to baseline serum levels and CEA, CYFRA and NSE reduction ≥ 20%

	Mean OS (95% CI)^a^ (months)	p-value
*Baseline CEA*		
< 5	12.2 (8.1–16.0)	0.035
≥ 5	5.6 (2.9–8.2)	
*Baseline CYFRA21-1*		
< 3.3	13.2 (11.0–14.3)	0.005
≥ 3.3	5.6 (3.4–7.7)	
*Baseline NSE*		
< 13.4	10.0 (6.2–13.7)	0.028
≥ 13.4	2.2 (0.2–5.0)	
*CEA reduction ≥ 20%* ^a^		
No	9.9 (8.5–11.3)	0.026
Yes	15.0 (12.7–17.3)	
*CYFRA21-1 reduction ≥ 20%* ^a^		
No	10.0 (8.4–11.6)	0.019
Yes	14.6 (12.4–16.8)	
*NSE reduction ≥ 20%* ^a^		
No	11.6 (9.9–13.4)	0.950
Yes	12.4 (9.8–15.0)	

^a^Median survival not reached

## Discussion

Immune checkpoint inhibitors such as anti-PD1 and anti PD-L1, are a recent option of treatment widely used for advanced cancers, including NSCLC. However, a substantial proportion of patients do not respond to these agents and display severe toxicities that lead to discontinuation of treatment [27]. On the other hand, in a small proportion of patients who do response, immunotherapy appears capable of producing long-term responses with substantial survival benefits [28]. For these reasons the discovery of biomarkers able to predict efficacy would be useful to select patients who might benefit from this therapy. Recently, particularly in melanoma cancer, several studies have investigated the association between routinely available peripheral blood biomarkers and response to immunotherapy [29–35]. Baseline or post-treatment changes in absolute leucocytes count (ALC), leucocytes sub-type counts, serum lactate dehydrogenase (LDH) and CRP levels, are among the most promising aim able to predict tumor response and survival in advanced melanoma patients treated with anti-PD-1 [30, 31] or anti-CTLA4 therapy [32–35]. Conversely, in advanced NSCLC, a few blood markers have been proposed as prognostic biomarkers for nivolumab therapy. In particular, higher baseline neutrophil to lymphocytes ratio (NLR) and platelet to lymphocyte ratio (PLR) have shown significant association with worse survival outcomes [36]. In addition, a recent study has examined a panel of six blood biomarkers showing as a combination of high ALC, high absolute eosinophil count (AEC) and low absolute neutrophil count (ANC) was associated with better survival outcome in NSCLC patients treated with nivolumab [37]. The role of CEA and CYFRA21-1 in monitoring tumor response during a first-line chemotherapy has been previously demonstrated in a publication from our Institution [8] and in a recent meta-analysis [38], but their role as predictive or treatment monitoring markers with immunotherapy has not yet been elucidated. To the best of our knowledge, this is the first study focusing on the role of CEA, CYFRA21-1 and NSE as potential markers for tumor response in advanced NSCLC patients treated with nivolumab. Interestingly, after 4 cycles of nivolumab, we observed that a CEA or CYFRA21-1 reduction ≥ 20% over the baseline was significantly associated with a better response (at least a disease control) whereas high baseline markers serum levels did not correlate with response to nivolumab. Multivariate analysis confirmed the positive association between CYFRA21-1 reduction and DCR. In addition previous studies in advanced NSCLC patients had showed that changes in CEA or CYFRA21-1 levels during chemotherapy [8, 38], radiochemotherapy [39] or targeted therapy [15, 16], had a higher predictive value than baseline level alone, indicating the usefulness of both markers for treatment monitoring. In agreement with these studies, we observed a reduction of the tumor markers > 20% already at the beginning of the therapy, in particular after the first two cycles, suggesting a possible role as markers able of monitoring the tumor response in an initial phase of the treatment also with immunotherapy. We also observed a good concordance between histological types and tumor markers. In adenocarcinoma and squamous cell carcinoma a CEA and a CYFRA21-1 reduction ≥ 20%, respectively, were significantly associated with a tumor response to nivolumab. In our study high baseline values of CEA and CYFRA21-1 were associated with worse OS, and, only for CEA, also with worse PFS. In this regard, data in literature are rather controversial. A recent study [40] reported as a pretreatment serum CYFRA21-1 level ≥ 2.2 ng/ml was an independent predictor of a favorable PFS (median PFS 155 vs 51.5 days, p = 0.05), while according to other authors [41] a baseline serum CEA level ≥ 5 ng/ml was associated with worse PFS. In our study multivariate analysis showed that normal baseline CEA or CYFRA21-1 levels and a more than 2 prior lines of therapies were independent prognostic factors in patients treated with nivolumab. These results suggest that NSCLC patients with normal pretreatment CEA or CYFRA21-1 test show a better OS. In addition, we observed a significant correlation between markers reduction after 4 cycles of nivolumab and survival outcome. In particular, a CEA or CYFRA21-1 reduction ≥ 20% was significantly associated with better PFS and OS. Specifically, in the multivariate analysis the CYFRA21-1 reduction ≥ 20% contributed significantly to the prediction of PFS and had a significant trend towards a positive prognostic factor. Interestingly, in patients with adenocarcinoma we observed a positive association between CEA or CYFRA21-1 reduction ≥ 20% and longer PFS whereas in patients with lung squamous carcinoma a CYFRA21-1 reduction ≥ 20% was statistically associated with better PFS. In both the histotypes similar median OS was observed whereas longer median OS was observed only for adenocarcinoma patients with a CEA reduction ≥ 20%. Therefore, CEA and CYFRA21-1 seem to have a better performance when monitoring adenocarcinoma patients, whereas the low number of squamous carcinoma patients did not allow to draw a conclusion in this sense. These results confirm the association of CEA with adenocarcinoma and of CYFRA21-1 with squamous carcinoma reported in previous studies [42, 43]. We are aware of the limitation of our study. This was a mono-centric study in which all consecutive patients were treated with nivolumab in an expanded access program. However, since to include the patients in this program the physicians were obligated to follow some inclusion and exclusion criteria, that have not allowed to treat all the patients with nivolumab, the risk of a patient selection bias cannot be excluded. Indeed, our study included a relatively homogeneous population with the majority of the patients stage IV, male and smokers. A strength of our study, is the mono-institutional approach that ensure that all the clinical and instrumental assessments and survival data (DCR, PFS and OS) as well as the laboratory analysis were performed consistently among all the patients before and during the treatment and data were not missed. The reduced number of patients and events in our study did not allow to draw definitive conclusions and for this reason further investigations are warranted. However, the correlation of the CEA and CYFRA21-1 reduction ≥ 20% with DCR and longer PFS was highly significant. If validated, these findings may be useful to physicians to make clinical decision; for example, nivolumab treatment may be stopped in patient without an evidence of a radiologic response and without CEA or CYFRA21-1 reduction ≥ 20% at this time-point, given their poor survival outcome and their extremely low probability of achieving a controlled disease. In conclusion, CEA and CYFRA21-1 may serve as realible markers of efficacy in NSCLC patients treated with nivolumab, either when considering the determination of the markers at baseline, or a markers reduction ≥ 20% after 4 cycles of nivolumab. On the contrary, the reduction of NSE was not significant for monitoring the efficacy of nivolumab. Further studies in a large population need to be conducted to confirm these results that may predict response and survival to immunotherapy.

## Conclusion

In summary, in advanced NSCLC patients we investigated the utility of analyzed three available serum tumor markers in predict tumor response and survival during the treatment with nivolumab. This is the first study that has analyzed the correlation between CEA, CYFRA21-1 and NSE reduction over the baseline and the tumor response. We found that a CEA or CYFRA21-1 reduction ≥ 20% after 4 cycles of nivolumab may serve as a reliable early marker of efficacy significantly associated with better DCR and PFS. Monitoring the changes in CEA or CYFRA21-1 during the treatment with nivolumab may be of great interest for the prediction of tumor response and survival.

## Abbreviations

AEC: absolute neutrophil count
ALC: absolute leucocytes count
ANC: absolute eosinophil count
anti-PD1: anti-programmed cell death 1
anti-PD-L1: anti-programmed cell death ligand 1
CEA: carcinoembryonic antigen
95%CI: 95% confidence intervals
CR: complete response
CRP: C-reactive protein
CT scan: computed tomography scan
CTLA4: cytotoxic T-lymphocyte antigen 4
CYFRA21-1: cytokeratin fragment 19
DCR: disease control rate
EGFR-TKIs: epidermal growth factor tyrosine kinase inhibitors
ECOG: Eastern Cooperative Oncology Group
EMA: european medicines agency
FDA: food and drug administration
HR: hazard ratio
LDH: serum lactate dehydrogenase
NLR: neutrophil to lymphocytes ratio
NOS: not otherwise specified
NSCLC: non-small cell lung cancer
NSE: neuron-specific enolase
OR: odds ratios
OS: overall survival
PD: disease progression
PD-1: programmed cell death 1
PD-L1: programmed cell death ligand 1
PFS: progression free survival
PLR: platelet to lymphocyte ratio
PR: partial response
PS: performance Status
RECIST: response evaluation criteria in solid tumors
SCLC: small-cell lung cancer
SD: stable disease
